# Falls prevention interventions for community-dwelling older people living in mainland China: a narrative systematic review

**DOI:** 10.1186/s12913-020-05645-0

**Published:** 2020-08-28

**Authors:** Pengpeng Ye, Yishu Liu, Jing Zhang, Ke Peng, Xuru Pan, Yang Shen, Shaoming Xiao, Elizabeth Armstrong, Yuliang Er, Leilei Duan, Rebecca Ivers, Lisa Keay, Maoyi Tian

**Affiliations:** 1 The George Institute for Global Health, University of New South Wales, PO Box M201, Missenden Road, Sydney, NSW 2050 Australia; 2 National Centre for Non-Communicable Disease Control and Prevention, Chinese Centre for Disease Control and Prevention, Beijing, China; 3 The George Institute for Global Health at Peking University Health Science Centre, Beijing, China; 4 School of Population Health, University of New South Wales, Sydney, Australia; 5 School of Public Health, The University of Sydney, Sydney, Australia; 6 School of Public Health, Peking University Health Science Centre, Beijing, China; 7 Falls Balance and Injury Research Centre, Neuroscience Research Australia, Randwick, Australia; 8 School of Optometry and Vision Science, University of New South Wales, Sydney, Australia

**Keywords:** Falls prevention, Interventions, Older people, Community, China

## Abstract

**Background:**

Falls in community-dwelling older people have been recognised as a significant public health issue in China given the rapidly growing aged population. Although there are several reviews documenting falls prevention programs for community-dwelling older adults, no systematic reviews of the scope and quality of falls prevention interventions in Mainland China exist. Therefore, the aim of this study was to systematically review falls prevention interventions for community-dwelling older people living in Mainland China.

**Methods:**

We systematically reviewed literature from Chinese and English databases. All types of randomised controlled trials (RCTs) and quasi-experimental studies published from 1st January 1990 to 30th September 2019 were included. Observational studies and studies in care facilities and hospitals were excluded. Narrative synthesis was performed to summarise the key features of all included studies. Quality assessment was conducted using the Cochrane Risk of Bias Tool and ROBINS-I tool for randomised and non-randomised studies respectively.

**Results:**

A total of 1020 studies were found, and 101 studies were included in the analysis. Overall, very few high quality studies were identified, and there was insufficient rigor to generate reliable evidence on the effectiveness of interventions or their scalability. Most interventions were multiple component interventions, and most studies focused on outcomes such as self-reported falls incidence or awareness of falls prevention.

**Conclusion:**

There is an opportunity to undertake an evaluation of a rigorously-designed, large-scale falls prevention program for community-dwelling older people in Mainland China. To help mitigate the rising burden of falls in Mainland China, recommendations for future falls prevention interventions have been made. These include: (1) target disadvantaged populations; (2) incorporate personalised interventions; and (3) investigate the effectiveness of those under-explored interventions, such as psychological, social environment, management of urinary incontinence, fluid or nutrition therapy and surgery. The study results will also potentially provide a useful evidence base for other low-and-middle income countries in a similar situation.

## Background

Falls, defined as ‘events which result in a person coming to rest inadvertently on the ground or floor or other lower level’ [1], are a major health issue for older adults [2]. Globally, about 30% of community dwelling people aged 65 years and over will fall at least once each year [2]. This number increases to 50% for those older than 85 years [2]. The Global Burden of Disease (GBD) Study 2017 estimated falls as the 11th cause of all-cause disability-adjusted life-years (DALYs) and they caused 32 million severe injuries and 400,000 deaths for people aged 70 years and above worldwide [3]. In China, falls are one of the leading causes of injury-related DALYs amongst older people in 2017 [4]. The GBD 2017 showed an increased incidence of falls and associated mortality and DALYs amongst older people in China over the past three decades [4].

Falls have been widely recognised as a complex but preventable health issue amongst older people [3]. A variety of community-based falls prevention interventions have been documented and evaluated in recent reviews [5–13]. Exercise interventions have been shown to be effective in reducing fear of falling and falls amongst community-dwelling older people [8, 9, 13]. The combination of exercise, education, medication modification and home environment improvements have also been found to be an effective packaged intervention [6, 7, 10–12]. However, most studies included in these reviews were conducted in high income countries. Few studies conducted in the Mainland China were synthesized in the previous reviews [11, 12]. The scope and quality of falls prevention interventions for community-dwelling older people in Mainland China also remain unclear. Therefore, the aim of this study was to systematically review falls prevention interventions for community-dwelling older people in Mainland China, to help inform policy and practice in a country with a rapidly rising burden of falls.

## Methods

### Protocol and registration

The protocol of this systematic review was registered in PROSPERO (CRD42018085507). This review is reported according to the guidance of the Preferred Reporting Items for Systematic Review and Meta-analysis (PRISMA) [14].

### Search strategy and study selection

A systematic search of the literature in both Chinese and English published from 1st January 1990 to 30th September 2019 was performed using the following electronic databases: Medline, Excerpta Medica Database (Embase), Web of Science, Cumulative Index to Nursing and Allied Health Literature (CINAHL), Cochrane Library, Chinese National Knowledge Infrastructure (CNKI), Chinese Wanfang Database, China Biology Medicine disc (CMBdisc), and Chongqing VIP Database for Chinese Technical Periodical. The searched keywords included fall, accidental falls, aged, senior, elderly, old, older, community, intervention, prevention, China, and Chinese. A detailed search strategy is listed in Additional File 4. The reference lists of included studies were scanned for additional potential studies. Two independent reviewers (PY and YL) screened the search results according to the eligibility criteria. Disagreements were resolved through discussion and a third reviewer (JZ) where necessary.

### Eligibility criteria

We included all articles reporting falls prevention interventions for community-dwelling older people in Mainland China. All types of randomised controlled trials (RCTs) and quasi-experimental studies with or without comparators were included. We only included studies satisfying the following criteria: (1) study participants aged 60 years and above with autonomous mobility or with adequate mobility when using assistive devices; and (2) conducted in community settings in Mainland China. We excluded studies conducted in care facilities or hospitals and/or non-peer reviewed research articles. There was no restriction applied to study outcomes.

### Data extraction

A standardised data abstraction form was developed (Additional File 1) for entering key study characteristics, including language, location, area, year, study population, design, protocol, intervention duration, intervention type, comparator, outcome measurements, and results. Two reviewers (PY and YL) independently extracted the data into the form and cross-reviewed. Disagreements were resolved by consensus.

### Quality assessment

For RCTs, methodological quality was assessed using the Cochrane Risk of Bias Tool [15]. We assessed the random sequence generation, allocation concealment, blinding of participants, personnel and outcomes assessors, incomplete outcome data, selective outcome reporting, and other sources of bias. Non-randomised trials were assessed by the ROBINS-I tool (Risk of Bias in Non-Randomised Studies – of Interventions) [16]. Any discrepancies were discussed and resolved by consensus (PY, YL and JZ).

### Data synthesis

We utilised a slightly adapted classification of falls prevention interventions from Hopewell’s Cochrane review [10]. In this review, the interventions were classified into three types (single intervention, multiple component intervention and multifactorial intervention) and nine categories (exercise, medication, surgery, management of urinary incontinence, fluid or nutrition therapy, psychological intervention, environment/assistive technology, social environment and knowledge/education). In each category, there were different intervention components (Additional File 2). Single intervention type is comprised of one or more intervention component from single intervention category alone. Multiple component intervention type provides a fixed combination of two or more fall prevention components from different intervention categories. Multifactorial intervention type provides people with an individualised combination of two or more components from different intervention categories based on assessment of modifiable risk factors. The study outcomes were categorized into four types based on the information extracted from all eligible studies, including fall-related injuries, fall incidence, fall-related physical performance and other outcomes related to falls (Additional File 3). A quantitative meta-analysis was not performed due to the heterogeneity of the included studies.

## Results

### Study selection

**Fig. 1:**
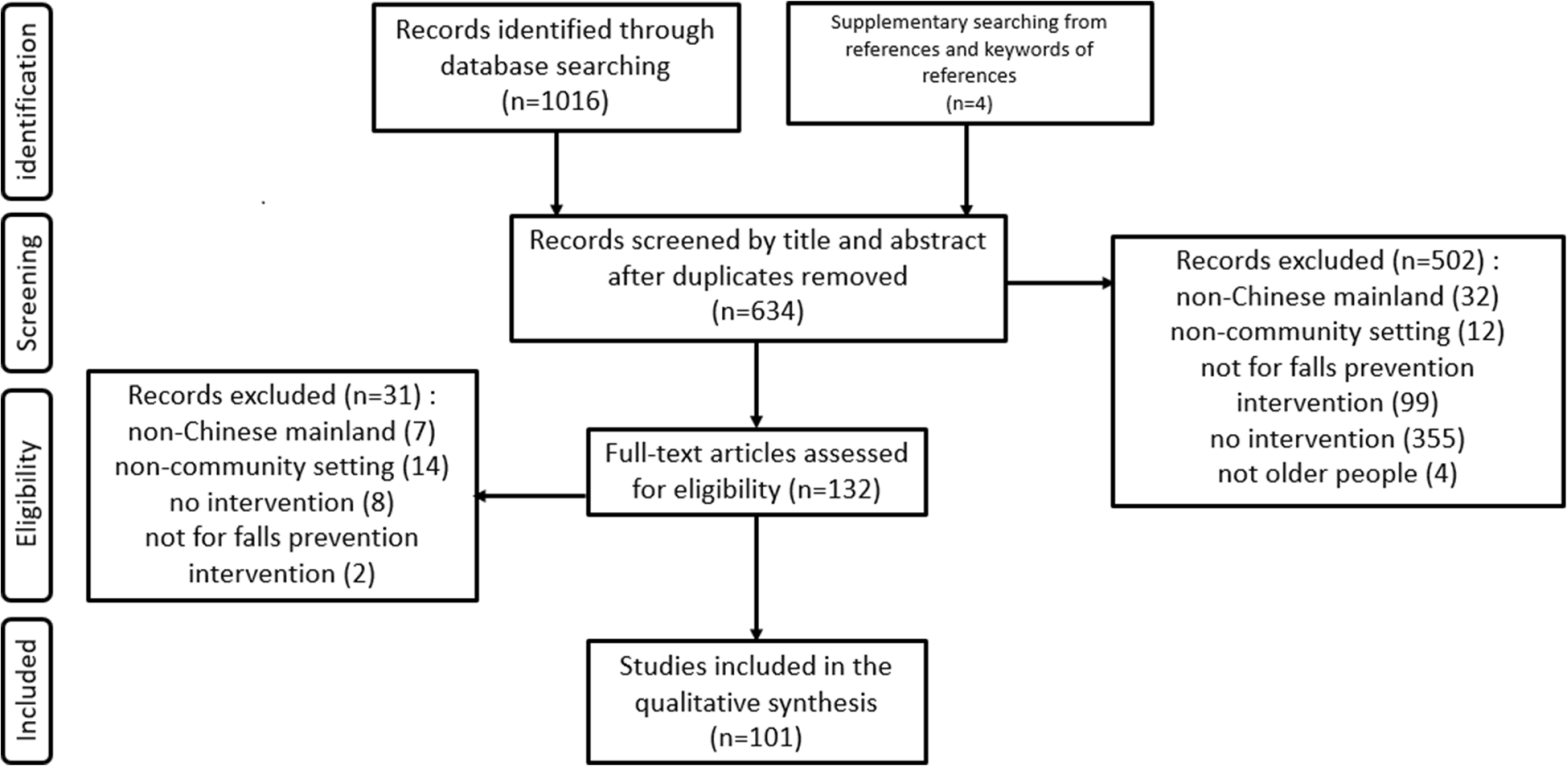
Flow diagram literature of search and review

We retrieved 1020 articles from nine databases and 132 articles were selected for full-text review (Fig. 1). Of those, 31 articles were excluded and 101 articles were included in the final synthesis [17–117].

### Study characteristics

**Fig. 2:**
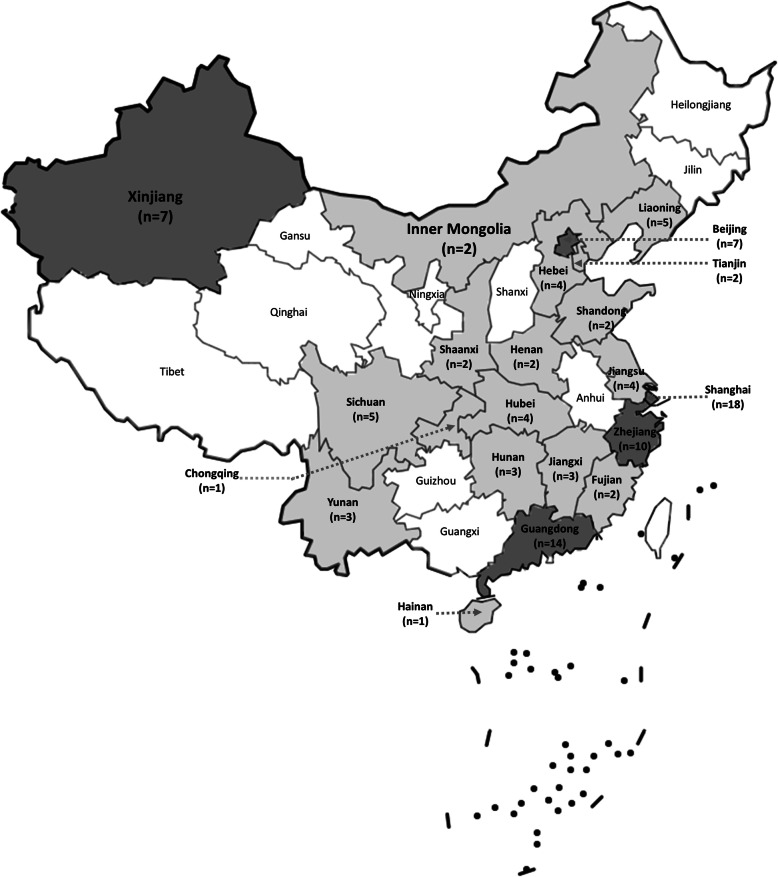
The number of studies in 31 provinces of Mainland China^*^ (^*^Provinces with seven or more studies are in dark grey)

The study characteristics are summarised in Additional File 1. All 101 studies were published after the year 2000 with 68 studies conducted since 2011. Studies were conducted across 21 Chinese provinces. Almost half of the studies were conducted in high socioeconomic regions: 18 studies in Shanghai municipality, 14 studies in Guangdong province, 10 studies in Zhejiang province, and 7 studies in Beijing municipality (Fig. 2). The majority of studies (97 studies) were conducted in urban settings with only 4 studies focusing on a rural population. There were 77 studies with RCT design and 24 studies with quasi-experimental design. The sample size of the RCT studies varied from 22 participants to 2643 participants, and in the quasi-experimental studies, sample size varied from 16 participants to 3466 participants. Only 4 RCTs and 4 quasi-experimental studies had sample sizes of more than 500 participants.

Single interventions were tested in 17 RCTs and 3 quasi-experimental studies, whereas multiple component interventions were tested in 47 RCTs and 15 quasi-experimental studies. Multifactorial interventions were tested in 13 RCTs and 6 quasi-experimental studies (Table 1, Additional File 2). Most studies reported two or more outcomes. The majority of outcomes were falls per person and falls prevention awareness (Table 2, Additional File 3).

**Table 1:** Number of studies by intervention type and study design

Study design	Single intervention			Multiple component intervention			Multifactorial intervention			Total
	Education	Exercise	Social environment	2–3 components	4–5 components	≥6 components	2–3 components	4–5 components	≥6 components	
RCT	2	14	1	16	30	1	6	6	1	77
Quasi-experimental	1	2	0	8	7	0	3	2	1	24
Total	3	16	1	24	37	1	9	8	2	101

**Table 2:** Number of studies^*^ by outcome and study design

Study design	Fall-related injuries			Fall incidence		Fall-related physical performance							Other outcomes related to falls			
	Facture events per person	Hospital admissions per person	Medical cost	Fallers per person	Fall events per person	Balance ability	The risk of falling	Gait	Flexibility	Muscle strength	Reaction time	Function ability of sensors	Prevention awareness	Quality of life	Environmental risk	Psychological risk
RCT	7	1	1	48	11	17	11	6	6	6	4	1	36	8	4	6
Quasi-experimental	1	1	0	19	1	2	1	1	1	0	0	0	13	1	5	1
Total	8	2	1	67	12	19	12	7	7	6	4	1	49	9	9	7

*One study may contain two or more outcomes

### Intervention types

#### Single intervention

The most commonly used single intervention category (16 studies) was exercise. In this category, the two most commonly used components were gait exercise, balance and functional training (10 studies), and strength/resistance exercises (9 studies). Traditional Chinese Health Qigong as three-dimensional training component, e.g., Tai Chi and Ba Duan Jin, was also adopted in 7 studies. There was only one RCT reporting the effectiveness of homecare services from social environment category to prevent falls for older people. There were two RCTs and one quasi-experimental study that evaluated knowledge/education intervention category (Additional File 2).

#### Multiple component intervention

In total, there were 63 studies utilising multiple component interventions. The number of intervention categories varied between two and six. Most studies (38 studies) adopted four or more categories. The combination of knowledge/education, exercise, environment/assistive technology and medication were the most commonly used package, adopted in 32 studies. The most common component for knowledge/education category was the distribution of health education written material (50 studies) and delivery of health lectures (49 studies). For exercise interventions, gait, balance and functional training (22 studies), strength/resistance exercises (11 studies) and three-dimensional training (11 studies) were usually adopted to prevent falls for older people. Furnishings and adaptations to homes and other premises (28 studies) were widely used in the environment/assistive technology category. There were 20 studies reporting the use of vitamin D and calcium supplementation as main components of medication category. As for the remaining five intervention categories, psychological and social environment were adopted in 15 and 5 studies, whereas surgery, management of urinary incontinence and fluid or nutrition therapy were not implemented in any of the included studies (Additional File 2).

#### Multifactorial intervention

Multifactorial interventions were the least commonly used intervention type (19 out of 101 studies). Up to 6 intervention categories were delivered to older people based on the assessment of their fall-related risk factors, including history of disease, history of falls, balance, fear of falling, home environment, and activities of daily living. The most commonly fixed combination of intervention categories was knowledge/education, environment/assistive technology and exercise. The main components in these three categories were very similar to those found in the multiple component intervention type, namely, the distribution of health education written material (16 studies) and delivery of health lectures (15 studies) in knowledge/education, furnishings and adaptations to homes and other premises (11 studies) in environment/assistive technology, and the gait, balance and functional training (3 studies) and three-dimensional training (3 studies) in exercise. Compared to the main components of the medication category in the multiple component type, the frequently used components of medication in multifactorial studies were medication withdrawal (4 studies) and dose reduction or increase, substitution or provision (3 studies). There were 9 studies reporting the use of a psychological intervention category, of which the most common component was an individual cognitive (behavioural) intervention (6 studies). Social environment category was utilized in 7 studies, of which staff training (5 studies) and service model change (studies) were the main components. Surgery, management of urinary incontinence and fluid or nutrition therapy categories were not adopted in any of the multifactorial intervention studies (Additional File 2).

### Outcomes

There were no studies reporting fall-related mortality as the study outcome. Fall-related injuries as outcomes of effectiveness of interventions were rarely reported, only for fall-related fracture events (8 studies), hospital admissions due to falls (2 studies) and medical cost (1 study). Fallers per person as a preferred indicator of fall incidence were reported in 67 studies, which was much more than studies reporting fall events per person (12 studies). As for fall-related physical performance, balance was one of most relevant causes of falls and reported in 19 studies, followed by risk of falling (12 studies), gait (7 studies), flexibility (7 studies), muscle strength (6 studies), reaction time (4 studies), and function ability of sensors (1 study). Other outcomes associated with the risk of falls were reported in some studies, of which 49 studies evaluated the awareness of falls prevention (49 studies), followed by quality of life (9 studies), environmental risk (9 studies), and psychological risk (7 studies). A wide range of measurement tools were used to assess the effect of the intervention, including standardised scales, questionnaires and devices. Despite the heterogeneity of outcome measures, all studies showed positive results (Additional Files 1 and 3).

### Risk of bias within and across studies

Risk of bias within and across RCTs is presented in Table 3. Most RCTs had a high risk of bias. Table 4 shows the risk of bias within and across quasi-experimental studies. There was a consistent pattern of moderate risk at pre-intervention, post-intervention and within studies.

**Table 3:** Risk of bias within and across RCTs

ID	Authors	Selection bias	Performance bias	Detection bias	Attrition bias	Reporting bias	Within study
1	Li W, et al. [18]	Unclear	High	Unclear	High	High	High
2	Duan F, et al. [24]	High	Unclear	Unclear	Low	Low	Unclear
3	Deng YN, et al. [25]	Unclear	High	Unclear	Low	Low	High
4	Zhang C, et al. [30]	Unclear	Unclear	Unclear	Low	Low	Unclear
5	Wang YH, et al. [31]	Unclear	High	High	Unclear	High	High
6	Feng MM, et al. [32]	Low	High	Low	Low	High	High
7	Mao XR, et al. [33]	Low	Unclear	Unclear	Low	High	High
8	Zhang CG, et al. [35]	Unclear	High	Unclear	Low	Low	High
9	Xie Y, et al. [36]	Unclear	Unclear	Unclear	Low	Low	Unclear
10	Liu YC, et al. [37]	High	High	Unclear	High	Low	High
11	Yang SL, et al. [40]	Unclear	Unclear	Unclear	Low	Low	High
12	Tang LJ, et al. [41]	High	High	Unclear	Low	High	High
13	Zheng WJ, et al. [42]	Unclear	Unclear	Unclear	Low	Low	Unclear
14	Xie XF, et al. [43]	High	Unclear	Unclear	Low	Low	High
15	Ren HJ, et al. [44]	Unclear	Unclear	Unclear	Unclear	Low	Unclear
16	Liu XY, et al. [45]	Unclear	High	Unclear	Low	Low	High
17	Zhan JH, et al. [46]	Unclear	High	Unclear	Low	Low	High
18	Guan SH, et al. [47]	Unclear	High	Unclear	Low	Low	High
19	Li YL, et al. [48]	High	High	Unclear	Low	Low	High
20	Liu LD, et al. [49]	Unclear	High	Unclear	Low	Low	High
21	Zhao D, et al. [52]	Low	Unclear	Unclear	Low	High	High
22	Zhou LL, et al. [53]	Unclear	Unclear	Unclear	Unclear	High	High
23	You MY, et al. [54]	High	Unclear	Unclear	Low	Low	High
24	Ou YB, et al. [55]	Unclear	High	Low	Low	Low	High
25	Tao WY, et al. [56]	Unclear	Unclear	Unclear	Low	Low	Unclear
26	Deng XW, et al. [57]	High	Unclear	Unclear	Unclear	High	High
27	Xiao Y, et al. [58]	Unclear	High	Unclear	Low	Low	High
28	Liu X, et al. [59]	Unclear	Unclear	Unclear	Low	Low	High
29	Ji YQ, et al. [60]	Unclear	High	Unclear	Low	Low	High
30	Zhang RL, et al. [61]	High	High	Unclear	High	Low	High
31	Li W, et al. [62]	High	High	Unclear	Unclear	Low	High
32	Chen CF, et al. [63]	Unclear	Unclear	Unclear	Low	Low	Unclear
33	Zhang J, et al. [64]	Low	Unclear	Unclear	Low	Low	Low
34	Mou YD, et al. [65]	High	Unclear	Unclear	Low	Low	High
35	Ma HX, et al. [66]	High	Unclear	Unclear	Low	Low	High
36	Li DY, et al. [67]	High	Unclear	Unclear	Unclear	High	High
37	Lu HL, et al. [69]	Unclear	Unclear	High	Unclear	High	High
38	Liang YY, et al. [70]	Unclear	High	Unclear	Low	Low	High
39	Li J, et al. [71]	Unclear	Unclear	Unclear	Low	Low	Unclear
40	Li H, et al. [72]	Unclear	High	Unclear	Low	Low	High
41	Li J, et al. [73]	Unclear	High	Unclear	Low	Low	High
42	Zeng YY, et al. [77]	Unclear	High	Unclear	Low	Low	High
43	Yuan Y, et al. [78]	Unclear	Unclear	Unclear	Low	Low	Unclear
44	Wang JY, et al. [79]	Unclear	High	Unclear	Low	Low	High
45	Qiu X, et al. [80]	Unclear	Unclear	Unclear	Low	Low	High
46	Liao SH, et al. [81]	Low	Low	Unclear	Low	Low	Unclear
47	Wu YQ, et al. [82]	Unclear	High	Unclear	Low	Low	High
48	Yun FX, et al. [83]	Unclear	High	Unclear	Low	Low	High
49	Tao YL, et al. [85]	Unclear	High	Unclear	Low	Low	High
50	Meng FY, et al. [86]	Unclear	High	Unclear	Unclear	Low	High
51	Wang DZ, et al. [87]	Unclear	High	Unclear	Unclear	Low	High
52	Tian YQ, et al. [88]	Unclear	High	Unclear	Low	Low	High
53	Yang XM, et al. [89]	High	High	Unclear	Low	Low	High
54	LanLi R, et al. [90]	High	High	Unclear	Low	Low	High
55	Ba HB, et al. [91]	Unclear	High	Unclear	Low	Low	High
56	Wu H, et al. [92]	Unclear	High	Unclear	Low	Low	High
57	Nu QL, et al. [93]	Unclear	High	Unclear	Low	Low	High
58	Yang Z, et al. [94]	Unclear	Unclear	Unclear	Low	Low	Unclear
59	Hu ZJ, et al. [95]	High	Unclear	Unclear	Low	Low	High
60	Qiu YY, et al. [97]	Unclear	High	Unclear	Low	Low	High
61	Wu ML, et al. [98]	Unclear	High	Unclear	Low	Low	High
62	Yang SL, et al. [99]	Unclear	High	Unclear	Low	Low	Unclear
63	Cai YB, et al. [100]	Unclear	High	Unclear	Unclear	Low	High
64	Yang JY, et al. [101]	Unclear	High	Unclear	Unclear	Low	High
65	Fan XY, et al. [103]	Unclear	Unclear	Unclear	Low	Low	Unclear
66	Gao R, et al. [104]	High	High	Unclear	Low	Low	High
67	Tang F, et al. [105]	High	High	Unclear	Unclear	Low	High
68	Hong D, et al. [106]	Unclear	High	Unclear	Low	Low	High
69	Zhao Y, et al. [108]	Unclear	High	Unclear	Unclear	Low	High
70	Wang K, et al. [109]	Low	Unclear	Unclear	Low	Low	Low
71	Xia QH, et al. [110]	Unclear	Unclear	Unclear	Low	Low	Unclear
72	Zhuang J, et al. [111]	Low	Unclear	Unclear	Unclear	Low	Unclear
73	Liu XH, et al. [113]	High	Unclear	Unclear	Low	High	High
74	Lv B, et al. [114]	Unclear	Unclear	Unclear	Low	Low	Unclear
75	Gao ML, et al. [115]	Unclear	Unclear	Low	Low	Unclear	Unclear
76	Deng XQ, et al. [116]	Unclear	Unclear	High	Unclear	High	High
77	Wu X, et al. [117]	Unclear	Unclear	Unclear	Low	Low	Unclear
*Overall across studies*		*Unclear*	*Unclear*	*Unclear*	Low	Low	*High*

**Table 4:** Risk of bias within and across quasi-experimental studies

ID	Authors	Pre-intervention	At-intervention	Post-intervention	Within study
1	Shi J, et al. [17]	Low	Unclear	Low	Moderate
2	Yan Q, et al. [19]	Moderate	Low	Moderate	Moderate
3	Yan YY, et al. [20]	Low	Low	Moderate	Moderate
4	Zhuang J, et al. [21]	Moderate	low	low	Moderate
5	Zhang LL, et al. [22]	Low	Low	Moderate	Moderate
6	Wan QP, et al. [23]	Low	Unclear	Moderate	Moderate
7	Sun XL, et al. [26]	Serious	Low	Unclear	Serious
8	GuLan DM, et al. [27]	Moderate	Low	Serious	Moderate
9	Wang J, et al. [28]	Low	Low	Serious	Serious
10	Ma CH, et al. [29]	Serious	Low	Unclear	Serious
11	Jiang CX, et al. [34]	Low	Unclear	Moderate	Moderate
12	Liu XY, et al. [38]	Moderate	Low	Low	Moderate
13	Chen LP, et al. [39]	Moderate	Unclear	Moderate	Moderate
14	Zhang X, et al. [50]	Moderate	Low	Moderate	Moderate
15	Mei D, et al. [51]	Low	Low	Moderate	Moderate
16	Zhou LW, et al. [68]	Unclear	Unclear	Moderate	Moderate
17	Jiang CY, et al. [74]	Moderate	Low	Moderate	Moderate
18	Guo YF, et al. [75]	Low	Low	Moderate	Moderate
19	Guo YF, et al. [76]	Moderate	Low	Low	Moderate
20	Hu SM, et al. [84]	Low	Low	Moderate	Moderate
21	Gui RL, et al. [96]	Moderate	Low	Moderate	Moderate
22	Guo LY, et al. [102]	Moderate	Low	Moderate	Moderate
23	Liu M, et al. [107]	Moderate	Unclear	Low	Moderate
24	Cao ZJ, et al. [112]	Low	Unclear	Low	Moderate
*Overall across studies*		Moderate	Low	Moderate	Moderate

## Discussion

In this review, we have reported on studies published in peer-reviewed journals that investigated falls prevention interventions for community-dwelling older people in Mainland China. We focused on reporting the types of interventions used as well as the components of falls prevention interventions. Although there are a growing number of studies in the past two decades focusing on this emerging public health issue in Mainland China, we identified a lack of research focused on rural areas. In addition, the quality of the evidence was generally poor, and studies did not address scalability and sustainability of interventions.

There were no studies identified prior to the year of 2000. Most studies were conducted after the year of 2011 and this aligns with the Chinese national policy development in relation to older people’s health management. The earliest policy attaching importance to falls prevention was the National Essential Public Health Service Package Specification (2009 Edition) issued by the National Health Committee on December 10, 2009 [118]. This policy specified older people’s health management as a universal health service provided to all age-eligible people. In addition, since 2009 there have been more than 22 relevant policies issued by Chinese ministries or departments affiliated to the State Council. Over the past decade, four national planning policies have been formulated by the Chinese central government to address the growing needs of the ageing population [119–122]. Of note, falls prevention for older people was specifically assigned as an important task in two policies, the 13th Five-Year Plan for Healthy Ageing (2016–2020) and Healthy China 2030 [121, 122]. There is evidence in the published studies of a strong alignment between the number of studies and the development of these policies. This suggests the prevention of falls has been identified by leading government agencies as a national key priority to achieving healthy ageing.

Nearly 50% of the studies were implemented in high socioeconomic urban areas. This may be explained by the local government in these areas providing researchers with better funding support to conduct falls prevention research, or, the higher education and health literacy levels of residents in urban areas providing researchers with greater access to, and recruitment of, participants in these areas compared to rural settings. Even when considering the change in population distribution between urban and rural areas since 2011 [123], the proportion of studies investigating community-based falls prevention interventions in rural areas was still disproportionately low given the rural population. This could imply that the impact of falls prevention interventions for community-dwelling older people was limited to the developed regions. Significant health inequalities might therefore occur in falls prevention services provided for community-dwelling older people in more rural provinces in Mainland China, particularly for resource-restricted areas.

In this review, we found falls prevention programs were predominately multiple component interventions, with a preferred combination of knowledge/education, exercise, environment/assistive technology and medication components. However, without taking the underlying risk of falling into consideration, the combination of interventions reported to be effective in studies investigating multiple component interventions might not be successfully applied to all older people in Mainland China due to differences in community settings. Additional evidence is required to demonstrate the effectiveness of these interventions in different community settings. Multifactorial interventions were recommended in the falls prevention guidelines [124–126], but they have not been widely implemented in the Chinese community settings. This result was consistent with the findings from Hopewell’s review that the lack of evidence of multifactorial interventions may be explained by the complexity of delivering these interventions, particularly with limited financial and supporting resources [10].

Among all intervention components, the top three frequently used components were distributing written materials, delivering lectures and providing gait, balance and functional training. The first two components were rarely implemented as standalone interventions. They usually acted as an integral part of the multiple component or multifactorial falls prevention bundles. Gait, balance and functional training was frequently adopted in all three types of falls prevention interventions, were found to be effective. This finding was consistent with previous studies [8, 9, 13]. In contrast, psychological, social environment, management of urinary incontinence, fluid or nutrition therapy, and surgery interventions were rarely or never used in all studies. There was also uncertain evidence of their effectiveness from the previous research. Their effectiveness in the Chinese community settings might be explored in the future.

Most studies reported two or more fall-related outcomes. Injuries, a harder outcome to measure, were rarely reported in the identified studies, but improved awareness after publicity of falls prevention information and education was reported in half of all 101 studies. Due to the large variation in outcome measures, a quantitative synthesis of the effect size, precision and variance of positive results reported in all selected studies was not performed, however our findings are consistent with the findings of other published literature [10–12]. None of the included studies provided a registered protocol and most were conducted with a small sample size. In addition, the level of bias assessed in these studies was high primarily due to the lack of information available within the randomized and non-randomized studies to assess bias. Despite all selected studies showed positive results, the interpretation of these results should be used with caution. Therefore, the high-quality evidence is currently not available to guide scale-up of the falls prevention interventions described in the studies. Thus, there is an opportunity to undertake an evaluation of a rigorously-designed, large-scale falls prevention program for community-dwelling older people in Mainland China.

This review synthesized the main characteristics of studies evaluating the effects of falls prevention interventions in community-dwelling older people in Mainland China. Three recommendations for future falls prevention research in Mainland China are: one, community-based falls prevention interventions need to be evaluated in rural settings; two, multifactorial interventions that include individual risk assessment and personalised interventions need to be investigated; three, the effectiveness of poorly utilised interventions, such as psychological, social environment, management of urinary incontinence, fluid or nutrition therapy and surgery, need further exploration and investigation for older people in Mainland China.

### Strengths and limitations

To our knowledge, this was the first review to systematically summarise the key features of studies delivering falls prevention interventions for community-dwelling older people living in Mainland China. This review provides an overview of the essential characteristics, interventions, and outcomes of studies conducted in community settings of Mainland China since 1990. Two limitations of this review have been identified Firstly, falls prevention interventions for community-dwelling older people in Mainland China might not have been identified by the search strategy since some interventions may be reported in non-peer-reviewed literature, e.g., reports, thesis or conference papers, and the characteristics of these interventions may differ from those reflected in this review. Secondly, a direct comparison and pooled analysis could not be performed due to the heterogeneity of outcome measurements.

## Conclusion

As population ageing has intensified, falls have been recognised as a preventable, but complex, health issue amongst older people. Community-based falls prevention interventions have been delivered to older people in different provinces of Mainland China during the last two decades. However, there is still a lack of evidence for robust, sustainable and scalable falls prevention interventions that could be integrated into policy and the health system in Mainland China. There is an urgent need to conduct well-designed studies to produce high-quality evidence for the most effective falls prevention interventions in Mainland China. The findings of this study will also potentially provide evidence base for other low- and middle-income countries with a rapidly ageing population but under-equipped health system to combat falls.

## Supplementary information

**Additional file 1.** The details of all eligible studies

**Additional file 2.** The types, categories and components of falls prevention of all eligible studies.

**Additional file 3.** The outcome types of all eligible studies.

**Additional file 4.** The example of detailed search strategy.

## Data Availability

All data analysed or produced as a result of this review are included in the main file and Additional files.

## Abbreviations

RCTs: Randomised controlled trials
ROBINS-I: Risk of Bias in Non-Randomized Studies–of Interventions
GBD: Global Burden of Disease
DALYs: Disability-adjusted life-years
PRISMA: Preferred Reporting Items for Systematic Review and Meta-analysis
Embase: Excerpta; Medica Database
CNKI: Chinese National Knowledge Infrastructure
CMBdisc: China Biology Medicine disc
