# Supplementary material for: Falls prevention interventions for community-dwelling older people living in mainland China: a narrative systematic review
Source: BMC Health Serv Res. 2020 Aug 28;20:808. doi: 10.1186/s12913-020-05645-0 (PMC7456050; doi:10.1186/s12913-020-05645-0)
Supplement: Supplementary file 4 — Additional file 4. The example of detailed search strategy. [file 12913_2020_5645_MOESM4_ESM.docx]

The example of detailed search strategy in Ovid Medline

| id | Searches | Results |
| --- | --- | --- |
| 1 | Accidental Falls/ | 22774 |
| 2 | fall*.tw. | 173381 |
| 3 | 1 or 2 | 179687 |
| 4 | exp China/ | 175182 |
| 5 | china*.tw. | 130472 |
| 6 | chinese.tw. | 181747 |
| 7 | 4 or 5 or 6 | 326084 |
| 8 | exp aged/ | 2996482 |
| 9 | old*.tw. | 1182949 |
| 10 | elder*.tw. | 217421 |
| 11 | senior*.tw. | 32625 |
| 12 | 8 or 9 or 10 or 11 | 3878723 |
| 13 | community*.tw. | 382606 |
| 14 | intervention*.tw. | 772946 |
| 15 | prevention*.tw. | 447894 |
| 16 | control*.tw. | 3169917 |
| 17 | 14 or 15 or 16 | 4019365 |
| 18 | 3 and 7 and 12 and 13 and 17 | 60 |
| 19 | limit 18 to (english language and yr="1990-2019") | 51 |

The example of detailed search strategy in CNKI

| 1. 跨库选择 | Step 1 Cross-library selection |
| --- | --- |
| 包含： 期刊 教育期刊 特色期刊 学术辑刊  不包含： 报纸 年鉴 专利 标准 成果 博士 硕士 国内会议 国际会议 | Libraries included: periodicals, education journals, characteristic journals, academic journals  Libraries excluded: doctoral and master's conferences, domestic conferences, international conferences, newspaper, yearbook, patent, standard, achievements |
| 2. 检索表达式 | Step 2 Search terms construction |
| SU=主题 AB=摘要  (SU='老年' or SU='老人' or SU='老年人' or SU='高龄') and (SU='跌倒' or SU='跌落' or SU='坠落' or SU='跌坠' or SU='跌跤' or SU='跌伤' or SU='绊倒' or SU='滑倒' or SU='滑落') and (SU='干预' or SU='预防' or SU='防止' or SU='防控' or SU='防治' or SU='防制') and (AB='社区' or AB='街道') | SU= topic AB= summary  (SU='laonian' or SU='laoren' or SU='laonianren' or SU='gaoling') and (SU='diedao' or SU='dieluo' or SU='zhuiluo' or SU='diezhui' or SU='diejiao' or SU='dieshang' or SU='bandao' or SU='huadao' or SU='hualuo') and (SU='ganyu' or SU='yufang' or SU='fangzhi' or SU='fangkong' or SU='fangzhi' or SU='fangzhi') and (AB='shequ' or AB='jiedao') |
| 3. 发表时间 | Step 3 Time period selection |
| 1990年1月1日-2019年9月30日 | 1^st^ January 1990 to 30^th^ September 2019 |
| 4. 结果 | Step 4 Results |
| 240 | 240 |
